# Criteria used to define tumor necrosis factor-alpha inhibitors failure in patients with moderate-to-severe psoriasis: a systematic literature review

**DOI:** 10.1080/07853890.2023.2192957

**Published:** 2023-04-04

**Authors:** Isabel Belinchon Romero, Almudena Mateu Puchades, Miguel Ribera Pibernat, Diana. P. Ruiz Genao, Pablo de la Cueva Dobao, Jose Manuel Carrascosa

**Affiliations:** a Department of Dermatology, Hospital General Universitario Dr. Balmis-ISABIAL, Alicante, Spain; b Department of Medicine, Universidad Miguel Hernández de Elche, Alicante, Spain; c Department of Dermatology, Hospital Universitario Doctor Peset, Valencia, Spain; d Department of Dermatology, Hospital Universitario Parc Taulí, Sabadell, Spain; e Department of Medicine, Universitat Autònoma de Barcelona, Barcelona, Spain; f Department of Dermatology, Hospital Universitario Fundación Alcorcón, Alcorcón, Spain; g Department of Dermatology, Hospital Universitario Infanta Leonor, Madrid, Spain; h Department of Dermatology, Hospital Universitari Germans Trias i Pujol, Badalona, Spain

**Keywords:** Psoriasis, tumor necrosis factor alpha (TNF-α), treatment failure, failure criteria

## Abstract

**Background:**

Determining tumor necrosis factor-alpha inhibitors (anti-TNF-α) failure is still a challenge in the management of moderate-to-severe psoriasis. Thus, our comprehensive systematic literature review aimed to gather information on the criteria used to define anti-TNF-α failure. We also aimed to discover the main reasons for anti-TNF-α failure and define subsequently administered treatments.

**Materials and methods:**

We conducted a systematic review following review and reporting guidelines (Cochrane and PRISMA). International (Medline/PubMed and Cochrane Library) and Spanish databases (MEDES, IBECS), and gray literature were consulted to identify publications issued until April 2021 in English or Spanish.

**Results:**

Our search yielded 58 publications. Of these, 37 (63.8%) described the criteria used to define anti-TNF-α primary or secondary failure. Criteria varied across studies, although around 60% considered Psoriasis Area and Severity Index (PASI)-50 criteria. Nineteen (32.8%) reported the reasons for treatment failure, including the lack or loss of efficacy and safety-related problems, mainly infections. Finally, 29 (50%) publications outlined the treatments administered after anti-TNF-α: 62.5% reported a switch to another anti-TNF-α and 37.5% to interleukin (IL)-inhibitors.

**Conclusion:**

Our findings suggest a need to standardize the management of anti-TNF-α failure and reflect the incorporation of new targets, such as IL-inhibitors, in the treatment sequence.

**KEY MESSAGES**
- In the treatment of psoriasis, the primary and secondary anti-TNF-α failure criteria differ widely in the scientific literature.
- The strictest efficacy criteria for defining anti-TNF-α failure, or those recommended by guidelines such as PASI75, were underused both in clinical trials and observational studies.
- Most studies failed to consider patient-reported outcomes in assessing psoriasis treatment efficacy, which contrasts with recent recommendations on the inclusion of patient-reported HRQoL as a supporting criterion when considering clinical outcomes.

## Introduction

Therapeutic options for moderate-to-severe psoriasis have dramatically increased in recent years due to the incorporation of new biologic agents. These include tumor necrosis factor-alpha inhibitors (anti-TNF-α) (etanercept, adalimumab, infliximab, certolizumab) and the interleukins (IL)-inhibitors (IL-12/23 [ustekinumab], IL-17 [secukinumab, ixekizumab, brodalumab, bimekizumab], IL-23 [guselkumab, tildrakizumab, risankizumab]) [1–4]. Choosing the appropriate treatment option for each patient can be challenging [3,5]. More recently, the availability of anti-TNF-α biosimilars has provided a less costly alternative to their precursors, facilitating the access to anti-TNF-α as a first-line biologic treatment for moderate-to-severe plaque psoriasis [6,7].

Notwithstanding, observations show that around 20% of patients discontinue the first course of anti-TNF-α and treatment discontinuation rates increase with time. The main reason for patients’ discontinuation seems to be a failure to respond or a loss of response to anti-TNF-α therapy [8]. Predicting or determining patients’ response to anti-TNF-α remains challenging in routine practice. This difficulty resides in the variety of clinical criteria followed, the multiple causes for treatment failure [9], and the lack of specific guidelines or recommendations on how to determine treatment failure or which biologic agent should be chosen after anti-TNF-α failure [9,10].

Therefore, there is a clear need to standardize the management of anti-TNF-α failure in patients with moderate-to-severe psoriasis. With this overarching objective in mind, we conducted the present systematic literature review, primarily to gather information on the criteria used to define treatment failure. Additionally, we investigated the main reasons for anti-TNF-α treatment discontinuation, the therapeutic options chosen after anti-TNF-α failure, the current recommendations on treatment choice after anti-TNF-α failure, and the main factors to consider when choosing a new therapy after anti-TNF-α failure in moderate-to-severe psoriasis patients. Taken together this information might provide us useful insights into current practices with a view to establishing the foundations for future standardization.

## Materials and methods

We conducted a systematic literature review following the Cochrane methodology [11] and reported our findings according to the Preferred Reporting Items for Systematic Reviews and Meta-Analyses (PRISMA) checklist for reporting [12] (see Supplementary Tables 1 and 2 for completed PRISMA checklists). This systematic approach minimized the risks of publication bias, ensuring the robustness of our results. To this end, we consulted international (Medline/PubMed and Cochrane Library) and Spanish national databases (Medicina en Español [MEDES], Índice Bibliográfico Español en Ciencias de la Salud [IBECS]). In addition, we undertook a broad search in the gray literature. The search targeted a wide range of publications, including original articles, systematic and narrative reviews, or clinical practice guidelines, published up until April 2021 in English or Spanish, focusing mainly on the criteria used to define anti-TNF-α failure in moderate-to-severe psoriasis patients. We also searched for the reasons why anti-TNF-α was discontinued in addition to the therapeutic options used after anti-TNF-α failure and the treatment recommendations for patients failing to respond to anti-TNF-α. Table 1 provides details of the publication inclusion and exclusion criteria, following the PICOTS (population, intervention, comparator, outcomes, time, and study design) definition.

**Table 1.** Eligibility criteria defined by PICOTS.

	Inclusion criteria	Exclusion criteria
Population	Patients with moderate-to-severe psoriasis	Patients with mild psoriasis or other dermatological conditions
Intervention (treatment)	Biological treatments based on anti-TNF-α	Other systemic treatments or biological agents other than anti-TNF-α
Comparator	N/A	
Outcome	Primary: Criteria used to define anti-TNF-α failure. Secondary: Reasons for anti-TNF-α treatment discontinuation. Therapeutic strategies after anti-TNF-α treatment failure. Current recommendations on choice of treatment after anti-TNF-α failure. Factors to consider when selecting a new therapy after anti-TNF-α failure	Efficacy and safety results Results obtained from biologic agents other than anti-TNF-α
Study Design	Original articles (including clinical trials and observational studies), reviews, meta-analyses, clinical practice guidelines, expert consensus	Opinion articles, letters to the editor, or conference communications
Time	Without limitation	N/A (not applicable)
Language	Published in English or Spanish	

Anti-TNF-α: tumor necrosis factor alpha inhibitors; N/A: not applicable; PICOTS: population, intervention, comparator, outcomes, time, and study design.

### Search strategies

To consult international and national databases, we used either free-text or Medical Subject Heading (MeSH) terms related to psoriasis, anti-TNF-α treatment, and treatment failure combined with Boolean operators (AND/OR) (see Supplementary Tables 3 and 4 for further details). Furthermore, we manually searched the reference lists of relevant articles obtained. Besides the database search, a broad gray literature search was also performed. Supplementary Table 5 describes the sources and search strategies applied for the gray literature.

Two researchers independently screened each of the identified publications. Any discrepancies between reviewers were resolved through consensus and, if necessary, by consulting a third reviewer. After duplicate removal by the EndNote reference manager (v. 9.X), publication screening was conducted in two phases. In the first phase, articles were screened by title and abstract according to the inclusion and exclusion criteria. In the second phase, articles selected for full-text reading in the previous phase were revised, considering the objectives of the review.

### Compliance with the reporting guidelines and level of evidence

Two researchers independently assessed the compliance of the original studies included to the reporting guidelines and the level of evidence of the publications. Any discrepancies were resolved by consensus and, if necessary, by consulting a third reviewer. To evaluate the compliance with the reporting guidelines, we applied the 30-point Consolidated Standards of Reporting Trials (CONSORT) statement for randomized clinical trials [13] and the 22 essential points of the Strengthening the Reporting of Observational Studies in Epidemiology (STROBE) declaration for observational cohort studies [14]. Studies were considered compliant when over 70% of items were reported. To evaluate the level of evidence of publications, we used the Oxford Centre for Evidence-Based Medicine (OCEBM) scales [15], which ranged from 1a (systematic reviews of randomized clinical trials) to 5 (expert opinion without explicit critical appraisal).

### Data abstraction

The variables recorded for the included articles comprised: the first author; the year of publication; the period of the study; the type of publication; the characteristics of the treatment and population; the outcomes evaluated; and their compliance with the reporting guidelines and level of evidence (see Supplementary Table 6 for further details).

## Results

### Results overview

**Figure 1.**
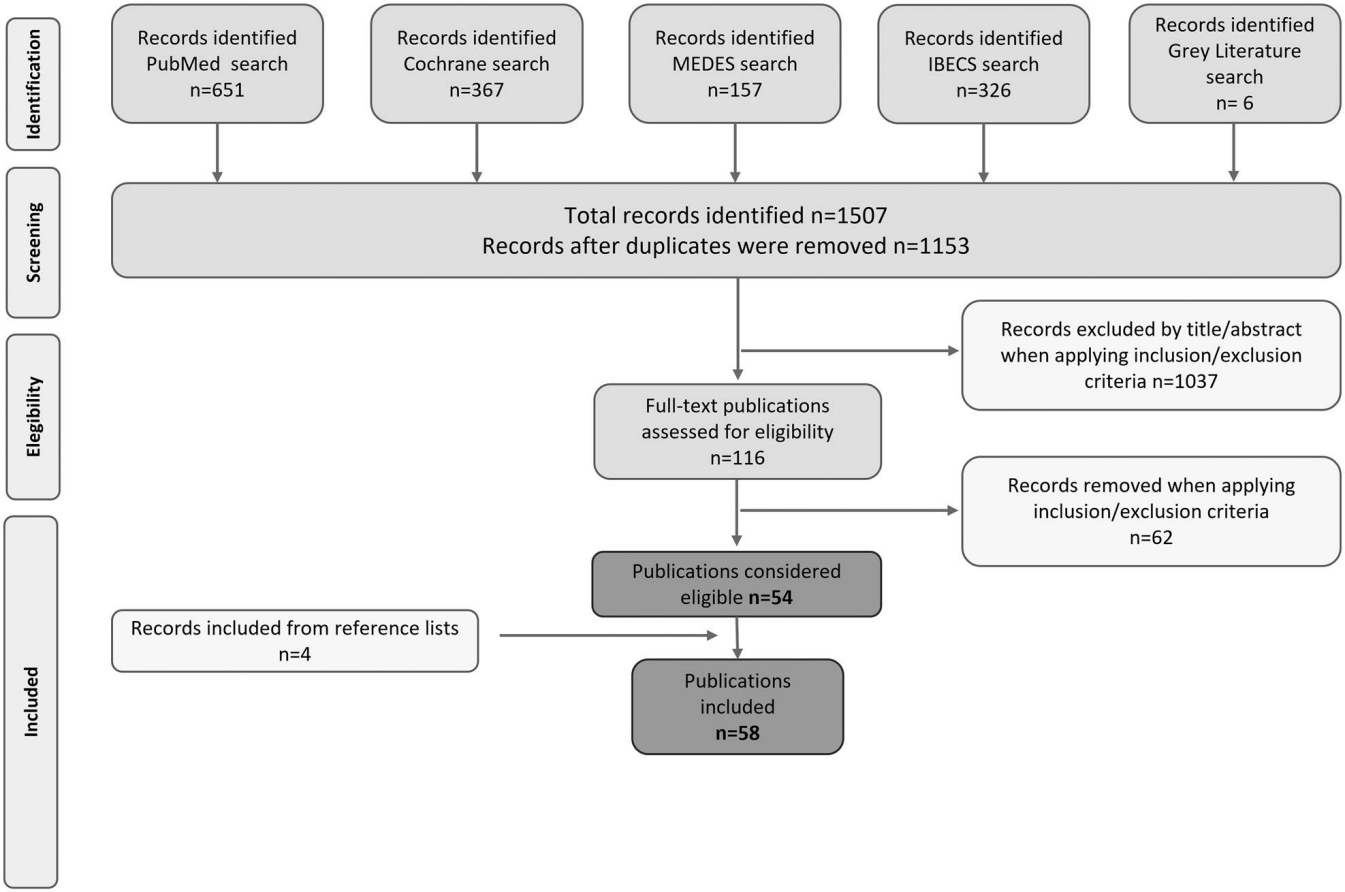
PRISMA flow diagram.

We retrieved 1507 publications in international (Medline/PubMed [*n* = 651], Cochrane Library [*n* = 367]) and Spanish databases (IBECS [*n* = 326] and MEDES [*n* = 157]) in addition to six publications from gray literature sources. After the removal of duplicates, a total of 1153 remained for title and abstract screening. Of these, 1037 were excluded in the first phase as they did not meet the eligibility criteria. We finally assessed 116 full-text articles, considering the review objectives, and identified 54 publications. Additionally, four further publications were recovered through the manual search of the reference lists of relevant articles (Figure 1). Thus, we finally obtained a total of 58 publications, of which 38 (65.5%) were observational studies, 16 (27.6%) were of clinical trial design, three (5.2%) were clinical practice guidelines, while one (1.7%) corresponded to a narrative review. Of the clinical trials (*n* = 16), we reviewed the CONSORT criteria in eight reports (50%) that had a randomized controlled design, of which six (75.0%) were compliant with the CONSORT reported items. Among the observational studies (*n* = 38), 23 (60.5%) had a retrospective design, whereas the rest (*n* = 15; 39.5%) followed a prospective design. We applied the STROBE criteria in 37 publications based on cohort observational designs and excluded a case-control study. Among the observational studies analyzed, 16 (43.2%) had good compliance with STROBE guidelines. Supplementary Table 7 provides a summary of all the studies included.

All the publications identified covered the main objective, and the secondary objectives stated. The following paragraphs describe the results obtained for each objective.

### Results by objective

#### Main objective: criteria used to define anti-TNF-α failure

We retrieved 37 (63.8%) publications—twenty-two observational studies (59.5%), 14 clinical trials (37.8%), and one clinical practice guideline (2.7%)—dealing with the current criteria used to define anti-TNF-α failure. Of these, 35 (94.6%) covered criteria for primary failure or lack of response, whereas 18 (48.6%) discussed criteria for secondary failure.

**Figure 2.**
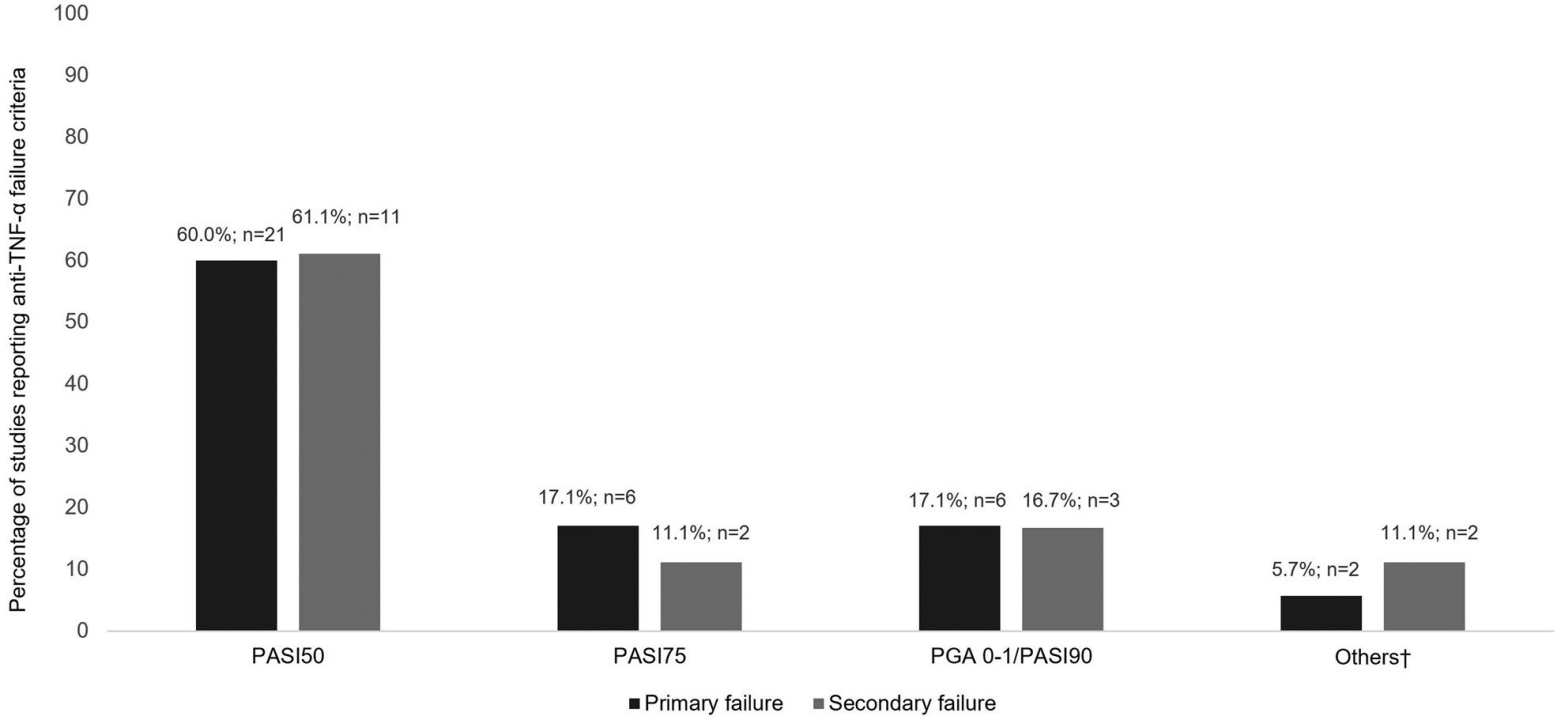
Distribution of studies according to the criteria applied for anti-TNF-α primary and secondary failure. Anti-TNF-α: tumor necrosis factor inhibitors; PASI: Psoriasis Area Severity Index; PGA: Physician’s Global Assessment; ^†^Non-objective clinical criteria or non-specified criteria.

**Figure 3.**
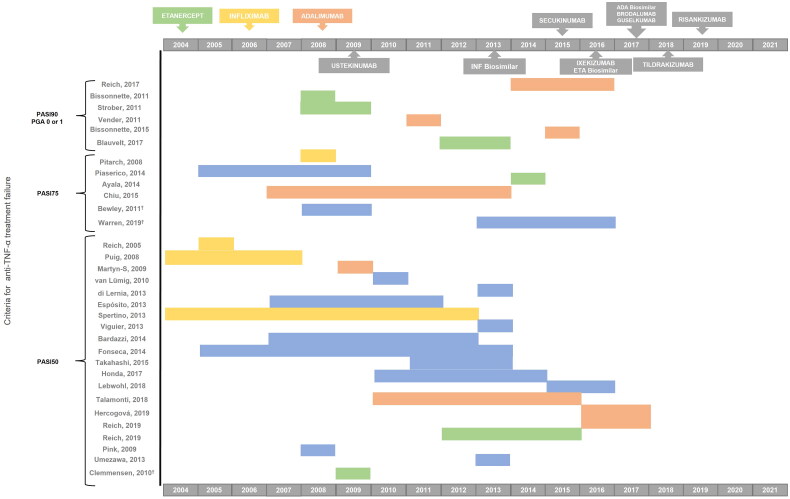
Criteria defining anti-TNF-α failure reported over time. The ordinate axis shows articles grouped by the standards of exigence of the criteria [from the strictest: failure to achieve PGA of 0 or 1 or a PASI90 to the least strict PASI50], whereas the abscissa axis shows the period in which they were conducted. Band colors represent the treatments for which the primary criteria for failure were described in the studies: green = etanercept; yellow = infliximab; red = adalimumab; blue = combination of anti-TNF-α. Abbreviations: ADA (adalimumab); anti-TNF-α (tumor necrosis factor inhibitors); ETA (etanercept); INF (infliximab); PASI (Psoriasis Area Severity Index); PGA (Physician’s Global Assessment). ^†^Includes patient-reported health-related quality of life (HRQoL) criterion too; ^‡^We did not include the IL-17 inhibitor, bimekizumab, since it had not yet been approved at the time of the study.

Regarding primary failure, most studies (*n* = 33; 94.3%) defined it using clinical criteria such as Psoriasis Area and Severity Index (PASI) or Physician’s Global Assessment (PGA). Nonetheless, the standards of these criteria varied from study to study, reporting most of them (*n* = 21; 60%) a less stringent PASI, inability to achieve a 50% reduction in PASI or PASI50. Figure 2 describes the distribution of the studies according to the criteria reported, whereas Supplementary Table 8 shows the details of the criteria reported in each study. Three of the studies (8.6%) included patient-reported health-related quality of life (HRQoL), measured by the Dermatology Life Quality Index (DLQI), in combination with clinical criteria to assess primary therapeutic failure. One of them defined treatment failure as not improving DLQI by 50% or below the value of 10 and/or failure to achieve PASI50 [16] while the other two considered it as a 5-point reduction in DLQI together with the failure to achieve PASI50 or PASI75 [17,18]. Apart from the variability of criteria, there was no clear pattern between the criteria and the period in which the studies were undertaken (Figure 3 shows the articles grouped together by the standards of the criteria [from the least strict, i.e. failure to achieve PASI50, to the strictest, namely a PGA of 0 or 1 or a PASI90] and the period in which they were conducted).

The standards of secondary failure (*n* = 18) criteria also varied from study to study. Loss of efficacy after reaching PASI50 was the most frequent criteria used (*n* = 11; 61.1%). Additionally, two publications (11.1%) did not specify the criteria used [19,20].

#### Secondary objectives

##### Main reasons for anti-TNF-α treatment discontinuation

We obtained 19 (32.8%) publications, specifically 17 (89.5%) observational studies and two (10.5%) clinical trials, which dealt with the main reasons for anti-TNF-α treatment discontinuation. Of these, sixteen also provided us detailed information to estimate the distribution of patients among the different causes for discontinuation. Accordingly, most anti-TNF-α discontinuations were due to efficacy-related reasons, mainly a lack or loss of efficacy or due to disease remission. The following reasons for discontinuation were safety-related and, more specifically, adverse events occurrence, with infections cited as the most commonly reported adverse event causing treatment withdrawal. Finally, other reasons for discontinuation that were not related to the treatment itself were patients’ preferences (e.g. patients’ willingness to be referred to a nearby hospital), lack of adherence to treatment, comorbidities unrelated to psoriasis, or a lack of patients’ follow-up. Table 2 shows the reasons grouped into four categories: inefficacy (lack or loss of efficacy), safety, remission, and other causes.

**Table 2.** Reasons for anti-TNF-α treatment discontinuation.

Study, year	Arnold. 2016^†^	Ayala. 2014	Bayaraa. 2019	Clemmensen. 2010	Dávila-Seijo. 2016	Espósito 2013	Fonseca. 2014	Gerdes. 2018	Gniadecki. 2011	Lunder. 2019	Menter. 2016	Ortonne. 2011	Piaserico. 2014	Puig. 2019	Roche. 2018	van der Reeck.2014	Range (%)
Patients on anti-TNF-α therapy	696	38	211	179	1298	650	35	472	747	893	754	282	4389	185	423	116	–
Patients who discontinued	117	7	126	29	974^†^	178	47	16	242	359^†^	294	195	105^‡^	68	305^†^	45	–
Efficacy-related^‡^ discontinuations *n* (%)	68 (59.1)	1 (14.3)	131 (62.9)	21 (72.4)	422 (43.3)	128 (71.9)	44 (93.6)	–	183 (75.6)	228 (63.5)	135 (45.9)	179 (91.8)	70 (66.7)	48 (70.6)	224 (73.4)	34 (77.5)	14.3%–93.6%
Remission-related^‡^ discontinuations *n* (%)	2 (1.7)	–	1 (0.5)	–	236 (24.2)	–	–	–	–	–	–	–	–	–	–	–	0.5%–24.2%
Safety-related^§^ discontinuations n (%)	36 (30.8)	3 (42.8)	16 (7.7)	5 (17.3)	132 (13.6)	29 (16.3)	–	7 (43.7)	30 (12.4)	63 (17.6)	31 (10.5)	16 (8.2)	35 (33.3)	5 (7.4)	55 (18.1)	14 (31.1)	7.4%–43.7%
Other reasons^¶^ *N* (%)	20 (17.1)	3 (42.9)	60 (28.9)	3 (10.3)	184 (18.9)	21 (11.8)	3 (6.4)	9 (56.3)	29 (12.0)	68 (19)	143 (48.7)	–	–	15 (22)	41 (13.5)	3 (6.7)	6.4%–56.3%

Anti-TNF-α (tumor necrosis factor inhibitors); ^†^Numbers and percentage refer to treatment courses or series instead of patients; ^‡^Efficacy: lack and loss of efficacy or disease remission; ^§^Safety: adverse effects and deaths; ^¶^Others: preferences (request to change hospital, patients who do not attend medical visits, patient decision), development of psoriatic arthritis, lack of follow-up, accessibility (economic), unrelated comorbidities, self-injection problems, swallowing problems, pregnancy or intention, lack of adherence, vaccination, surgery.

##### Therapeutic options used after anti-TNF-α failure in moderate-to-severe psoriasis patients

**Figure 4.**
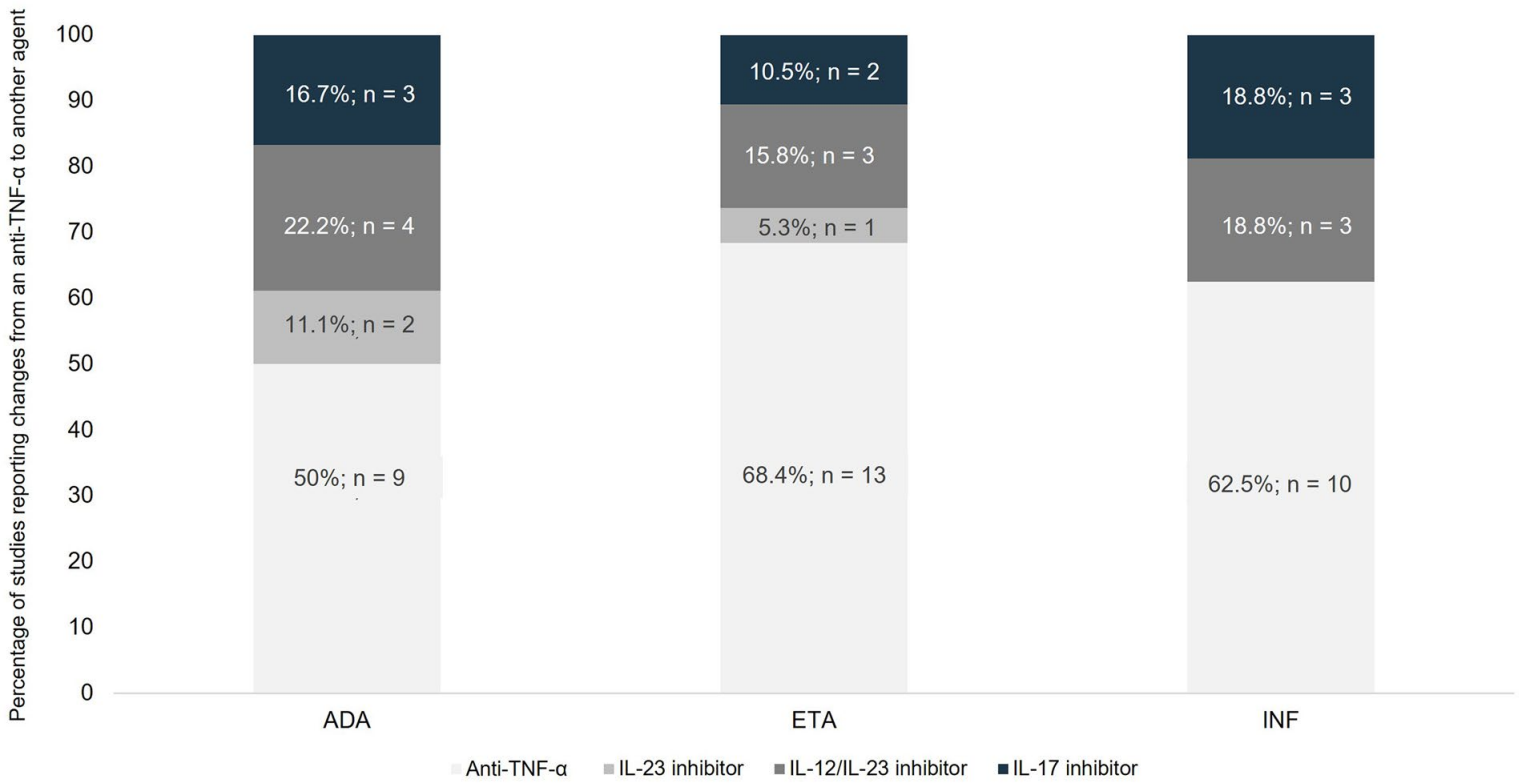
Therapeutic options after Anti-TNF-α loss of response: frequency of changes after etanercept, infliximab, and adalimumab failure. ADA: adalimumab; anti-TNF-α: tumor necrosis factor inhibitors; ETA: etanercept; IL: interleukin; INF: infliximab.

Twenty-nine publications (50%), including 17 (58.6%) observational studies and 12 clinical trials (41.4%), met the last objective: namely, they described the therapeutic options used after anti-TNF-α failure in moderate-to-severe psoriasis patients. Among these, we identified a total of 56 treatment changes after anti-TNF-α failure: most of these consisted in a shift to another anti-TNF-α (*n* = 35; 62.5%), while the others involved changes to an IL inhibitor (*n* = 21; 37.5%) mainly to an IL-12/IL-23 inhibitor (*n* = 10; 47.6%), followed by an IL-17 inhibitor (*n* = 8; 38.1%) or an IL-23 inhibitor (*n* = 3; 14.3%). When we considered the three anti-TNF-α evaluated—etanercept, infliximab, and adalimumab—separately, more studies showed a change of adalimumab to other therapeutic targets rather than other agents: for adalimumab, 50% (n/*N* = 9/18) of changes were to an IL inhibitor, compared with 31.6% (n/*N* = 6/19) for etanercept and 37.5% (n/*N* = 6/16) for infliximab. See Figure 4 for further details. Likewise, Supplementary Figure 1 reflects the treatment changes after anti-TNF-α failure reported over time, showing the other therapeutic targets to be adopted in the last ten years, such as IL-12/IL-23 inhibitors.

##### Current recommendations on the choice of treatment and the factors to consider when choosing a new therapy after anti-TNF-α failure

The recommendations on these issues were scarce and unspecific. Only four publications provided recommendations on treatment choices after anti-TNF-α failure, of which three (75%) were clinical practice guidelines from European settings [10,21,22] and one was a narrative review (25%) [23]. Of these, the French Society of Dermatology [21] was the only one proposing a treatment algorithm for patients with moderate-to-severe psoriasis and without significant comorbidities based on expert opinion. Moreover, one clinical practice guideline released by the British Association of Dermatologists [10] contained specific recommendations regarding which factors should be considered when choosing a new therapy after anti-TNF-α failure.

## Discussion

Anti-TNFs have become the predominant biological option to treat moderate-to-severe psoriasis patients due to the availability of biosimilars. However, determining anti-TNF-α failure to respond to these treatments and deciding the best alternative is one of the most challenging aspects of psoriasis management. Within this scenario, our systematic literature review aimed to cover key questions in the management of anti-TNF-α failure and, primarily, to know about the criteria used to define treatment failure and whether these criteria are uniform among studies.

The primary and secondary failure criteria differed widely among the studies appraised; however, failure to achieve PASI50 was the most commonly cited criteria. The predominance of PASI50 in the studies implies that stricter criteria such as the PASI75, recommended by the European consensus in 2011 [24] as the standard of response assessment for moderate to severe psoriasis, were underused. Our findings also showed that less than 20% of studies regard the strictest criteria: PASI90 or PGA 0 or 1, both for primary and secondary treatment failure. Both criteria are equivalent and show a ‘clear’ or ‘almost clear’ disease status and have traditionally been considered too stringent to define treatment response. In this respect, the European EuroGuiDerm Guidelines [25] consider these stricter criteria (PASI90 or PGA 0 or 1) as the new standards given new biologic treatments enable patients to achieve them at higher rates [26,27]. In particular, the Spanish Psoriasis Group (GPS) of the Spanish Academy of Dermatology and Venereology (AEDV) [28] has recently recommended PASI90 as the clinically appropriate objective for biologic treatment and PASI100 as the optimal objective. It is also noteworthy that all the studies considered a relative PASI or PASI response (i.e. a PASI reduction between baseline and a specified treatment period by a percentage) rather than the absolute PASI value. Absolute PASI has also begun to be recognized as a feasible alternative outcome for psoriasis, especially in routine practice [29], as it is independent of the baseline values and also correlates with clinically meaningful measures and HRQoL [30]. On that basis, the GPS of the AEDV also considered a PASI ≤ 3 as a clinically appropriate objective for biologic treatment [28]. Some guidelines also recommend applying HRQoL as a supporting criterion to modify the treatment when the therapeutic PASI response improves between 50% and 75%. Accordingly, therapy should be changed if the DLQI indicates a poorer HRQoL (DLQI > 5) but can be continued if DLQI ≤ 5, which indicates a better HRQoL status [24,31]. The recent EuroGuiDerm Guidelines support the use of HRQoL through DLQI or the Skindex-29 or Skindex-17 instruments, together with an objective assessment of treatment response [25]. In this regard, it is noteworthy that only three publications [16–18] in our review considered patient-reported HRQoL as a therapeutic failure criterion in combination with clinical criteria.

Another important question raised in our review relates to the most common reasons for anti-TNF-α discontinuation. As expected, efficacy-related reasons were the most frequent. The most-reported included inefficacy in terms of both lack and loss of efficacy. However, some studies include positive causes, such as remission of psoriasis symptoms, which accounted for up to 24% of the reasons for discontinuation [32]. The second most frequently reported reasons for treatment withdrawal were safety-related, specifically, infections. In this regard, there is conflicting evidence, with some studies showing an increase in infections with anti-TNF-α [33] and others suggesting that the risk of infectious episodes for biologics does not exceed the risk observed for conventional treatments [32,34]. Apart from tolerability, our results showed that continuance of the anti-TNF-α treatment might also be influenced by other factors unrelated to the drug itself. These include patient preferences, lack of patients’ follow-up, comorbidities, psoriatic arthritis, or lack of patients’ adherence. In this respect, prior literature indicates that discontinuation or switching of anti-TNF-α agents for nonmedical reasons (unrelated to clinical efficacy or tolerability) might result in worse clinical outcomes and increased health-care resource utilization [35,36].

With respect to the biologic agents chosen after anti-TNF-α failure, our findings reflect a change in treatment sequence in the last ten years, incorporating new targets, mainly IL-inhibitors. Among these, the IL-12/IL-23 inhibitor was predominantly chosen, followed by IL-17 inhibitors. These changes in the chosen treatment are not surprising as they reflect the chronological treatment approval sequence [37] and are supported by the latest clinical evidence [38]. Another aspect to consider is that for the three anti-TNF-α—etanercept, infliximab, and adalimumab—considered separately, we observed that a comparatively higher proportion of patients receiving adalimumab changed to non-anti-TNF-α targeted therapies. These differences might be because the studies were conducted in different time periods, and adalimumab was the last anti-TNF-α to receive approval, i.e. it was approved after etanercept and infliximab. The fact that adalimumab was the last anti-TNF-α to be approved increases the likelihood of a change from adalimumab to other agents such as ustekinumab rather than to another anti-TNF-α. This change also indicates a paradigm shift in favor of the IL-inhibitors, which have been shown more effective in terms of PASI75 and PASI90 scores than anti-TNF-α agents [39]. This fact also reflects physicians’ treatment preferences as they prioritize efficacy outcomes, such as the proportion of patients achieving a certain outcome (e.g. PASI90), considering these to be the most important factors for decision-making in lieu of characteristics like dosing attributes or route of administration [40–42]. However, it is important to keep in mind that incorporating individual patient preferences and characteristics in the selection of the optimal treatment favors greater adherence and satisfaction with the treatment [43,44]. In fact, it has previously been shown that demographic and socioeconomic characteristics, as well as the psychosocial burden of the disease, influence treatment decision-making [44–46].

### Limitations and strengths

We wish to describe certain limitations and strengths related to the design of the current review. With respect to the limitations, we acknowledge that we appraised a wide range of publications, including those with low reporting quality or a low level of evidence, such as narrative reviews. We recognize that the latter might introduce the author’s bias, which might likewise affect the results and conclusions of our study. Additionally, the inclusion criteria were limited to English and Spanish publications, possibly resulting in the omission of critical publications in other languages. Also, we are aware that treatment options for psoriasis continue to increase rapidly and, therefore, the validity of our results could be limited in time. For example, the IL-17 inhibitor, bimekizumab, was approved at the time of the review, so we could not include it in the search. On the other hand, the articles reviewed correspond to studies addressed to evaluate original molecules and no biosimilars were included. This is because for the approval of biosimilars, extrapolation of data is allowed, so that in some cases biosimilars are approved for the treatment of psoriasis without having been directly studied in comparative trials for this pathology but for others [47]. In fact, the European Medicines Agency has recently declared that biosimilars are interchangeable with their reference products and can be substituted for each other once they are approved in the EU [48]. To avoid including partial information from trials with biosimilars in our review, we decided not to include them; nevertheless, our findings should also be extrapolated to these molecules. Likewise, psoriasis treatment options have varied greatly over the years, meaning that the older studies included did not contemplate all the therapeutic options currently available. However, including studies from different periods could also be considered a strength of our review as this has allowed us to observe the changing trends in the management of psoriasis patients after anti-TNF-α failure. Other strengths include the fact that we have conducted our review according to a systematic approach following a strict search strategy, article selection, and data extraction requirements. This systematic approach ensured the robustness of our results as we minimized the publication bias. In addition, we did not restrict the time or location of the publications, so the conclusions drawn are not confined to a particular population or subgroup of psoriasis patients and could therefore be extrapolated to the general moderate-to-severe psoriasis population.

## Conclusions

Our findings suggest the need to standardize the current management of anti-TNF-α failure in psoriasis patients. The appraised evidence illustrates significant heterogeneity in the criteria used to define anti-TNF-α failure. Among the various criteria, PASI50 was chosen in a high proportion of studies and was applied indistinctly of the study period, even though the stricter PASI75 has only been recommended in recent years and new biologic treatments allow patients to achieve PASI75 and even PASI90 or PGA 0 or 1. Another finding is that most studies failed to consider patient-reported outcomes in assessing psoriasis treatment, which contrasts with recent recommendations on the inclusion of patient-reported HRQoL as a supporting criterion when considering clinical outcomes. The reasons for anti-TNF-α discontinuation also varied widely among studies, with efficacy or safety-related issues being the most common. However, our results also suggest that other factors, such as patients’ treatment preferences, should be considered. Finally, although the recommendations on which treatment to choose after anti-TNF-α failure are scarce, our findings reflect a pattern in the last ten years, with greater incorporation of new targets, mainly IL-inhibitors such as IL-12/IL-23, IL-17, and IL-23 inhibitors in the treatment sequence for moderate-to-severe psoriasis patients.

## Supplementary Material

Supplemental Material

## Data Availability

There is no raw data associated with this systematic review. The authors confirm that the data supporting the findings of this study are available within the article and supplementary materials.
