# Supplementary material for: Criteria used to define tumor necrosis factor-alpha inhibitors failure in patients with moderate-to-severe psoriasis: a systematic literature review
Source: Ann Med. 2023 Apr 4;55(1):1335–45. doi: 10.1080/07853890.2023.2192957 (PMC10075489; doi:10.1080/07853890.2023.2192957)
Supplement: Supplemental Material [file IANN_A_2192957_SM9162.doc]

**Supplementary Material**

Supplementary Table 1. PRISMA checklist for abstracts

Supplementary Table 2. PRISMA checklist

Supplementary Table 3. List of search terms considered for the search

Supplementary Table 4. Search strategies

Supplementary Table 5. Gray literature sources

Supplementary Table 6. Description of the variables extracted in the included articles

Supplementary Table 7. Summary of the studies included

Supplementary Table 8. Criteria reported by each study for primary and secondary failure

Supplementary Figure 1. Treatment after anti-TNF-α failure reported over time

Supplementary Table 1. PRISMA checklist for abstracts[1]

| **Section and topic** | **Item #** | **Checklist item** | **Location where item is reported** |
| --- | --- | --- | --- |
| **Title** |  |  |  |
| Title | 1 | Identify the report as a systematic review. | Page 1.Title |
| **Background** |  |  |  |
| Objectives | 2 | Provide an explicit statement of the main objective(s) or question(s) the review addresses. | Page 1. Abstract: “we conducted a systematic review to gather information, primarily on the criteria used to define anti-TNF-α treatment failure in moderate-to-severe psoriasis patients” |
| Metho**ds** |  |  |  |
| Eligibility criteria | 3 | Specify the inclusion and exclusion criteria for the review. | Page 1. Abstract: “to identify publications issued until April 2021 in English or Spanish.” |
| Information sources | 4 | Specify the information sources (e.g., databases, registers) used to identify studies and the date when each was last searched. | Page 1. Abstract: “We consulted international (Medline/PubMed and Cochrane Library) and Spanish databases (MEDES, IBECS) in addition to the gray literature to identify publications issued until April 2021” |
| Risk of bias | 5 | Specify the methods used to assess risk of bias in the included studies. | Page 1. Review and reporting guidelines (Cochrane and PRISMA) were followed to minimize publication bias. |
| Synthesis of results | 6 | Specify the methods used to present and synthesise results. | - |
| **Results** |  |  |  |
| Included studies | 7 | Give the total number of included studies and participants and summarise relevant characteristics of studies. | Page 1. Abstract: “Our search yielded 58 publications. Of these, 37 (63.8%) described the criteria used to define anti-TNF-α failure… Nineteen (32.8%) reported the reasons for treatment failure” |
| Synthesis of results | 8 | Present results for main outcomes, preferably indicating the number of included studies and participants for each. If meta-analysis was done, report the summary estimate and confidence/credible interval. If comparing groups, indicate the direction of the effect (i.e. which group is favoured). | Page 1. Abstract: Although criteria varied across publications, around 60% considered … Finally, 29 (50%) publications outlined the treatments administered after anti-TNF-α: 62.5% reported a switch to another anti-TNF-α and 37.5% to interleukin (IL)-inhibitors. |
| **Discussion** |  |  |  |
| Limitations of evidence | 9 | Provide a brief summary of the limitations of the evidence included in the review (e.g. study risk of bias, inconsistency and imprecision). | - |
| Interpretation | 10 | Provide a general interpretation of the results and important implications. | Page 1. Abstract Overall, our findings illustrate a significant heterogeneity regarding the criteria used to define anti-TNF-α failure and the reasons for its discontinuation. |
| **Other** |  |  |  |
| Funding | 11 | Specify the primary source of funding for the review. | Title page |
| Registration | 12 | Provide the register name and registration number. | - |

Supplementary Table 2. PRISMA checklist [1]

| **Section and Topic** | **Item #** | **Checklist item** | **Location where item is reported** |
| --- | --- | --- | --- |
| **TITLE** | | |  |
| Title | 1 | Identify the report as a systematic review. | Page 1.Title |
| **ABSTRACT** | | |  |
| Abstract | 2 | See the PRISMA 2020 for Abstracts checklist. | See the Supplementary table 1 (PRISMA checklist for abstracts) |
| **INTRODUCTION** | | |  |
| Rationale | 3 | Describe the rationale for the review in the context of existing knowledge. | Page 2; Introduction. From: “Therapeutic options for moderate-to-severe psoriasis have dramatically increased” to “… the lack of specific guidelines or recommendations on how to determine treatment failure or which biologic agent should be chosen after anti-TNF-α failure.” |
| Objectives | 4 | Provide an explicit statement of the objective(s) or question(s) the review addresses. | Pages 2 and 3; Introduction. From: “…we conducted the present systematic literature review, primarily to gather information on the criteria used to define treatment failure” to “… and the main factors to consider when choosing a new therapy after anti-TNF-α failure in moderate-to-severe psoriasis patients.” |
| **METHODS** | | |  |
| Eligibility criteria | 5 | Specify the inclusion and exclusion criteria for the review and how studies were grouped for the syntheses. | Page 3. Methods: “The search targeted a wide range of publications, including original articles, systematic and narrative reviews, or clinical practice guidelines, published up until April 2021 in English or Spanish”    Table 1 (Eligibility criteria defined by PICOS) |
| Information sources | 6 | Specify all databases, registers, websites, organisations, reference lists and other sources searched or consulted to identify studies. Specify the date when each source was last searched or consulted. | Page 3. Methods: “We consulted international (Medline/PubMed and Cochrane Library) and Spanish databases (MEDES, IBECS) besides the gray literature to identify publications issued until April 2021 in English or Spanish.” |
| Search strategy | 7 | Present the full search strategies for all databases, registers and websites, including any filters and limits used. | Pages 3 and 4. Methods. Search strategy: From “We conducted a systematic review of the literature ….” to “The search strategy included English and Spanish terms such as psoriasis, anti-TNF-α, AND/OR “therapeutic failure”  Supplementary Tables 3, 4 and 5 |
| Selection process | 8 | Specify the methods used to decide whether a study met the inclusion criteria of the review, including how many reviewers screened each record and each report retrieved, whether they worked independently, and if applicable, details of automation tools used in the process. | Page 4. Methods. Data abstraction. From: “Two researchers independently screened each of the identified publications” to “In the second phase, articles selected for full-text reading in the previous phase were revised, considering the objectives of the review” |
| Data collection process | 9 | Specify the methods used to collect data from reports, including how many reviewers collected data from each report, whether they worked independently, any processes for obtaining or confirming data from study investigators, and if applicable, details of automation tools used in the process. | - |
| Data items | 10a | List and define all outcomes for which data were sought. Specify whether all results that were compatible with each outcome domain in each study were sought (e.g. for all measures, time points, analyses), and if not, the methods used to decide which results to collect. | Page 5. Methods. Data abstraction. From: “The following variables were extracted for the articles finally included” to “compliance with the reporting guidelines and level of evidence” |
|  | 10b | List and define all other variables for which data were sought (e.g. participant and intervention characteristics, funding sources). Describe any assumptions made about any missing or unclear information. | - |
| Study risk of bias assessment | 11 | Specify the methods used to assess risk of bias in the included studies, including details of the tool(s) used, how many reviewers assessed each study and whether they worked independently, and if applicable, details of automation tools used in the process. | Page 4. Methods. Compliance with the reporting guidelines and level of evidence |
| Effect measures | 12 | Specify for each outcome the effect measure(s) (e.g. risk ratio, mean difference) used in the synthesis or presentation of results. | - |
| Synthesis methods | 13a | Describe the processes used to decide which studies were eligible for each synthesis (e.g. tabulating the study intervention characteristics and comparing against the planned groups for each synthesis (item #5). | - |
|  | 13b | Describe any methods required to prepare the data for presentation or synthesis, such as handling of missing summary statistics, or data conversions. | - |
|  | 13c | Describe any methods used to tabulate or visually display results of individual studies and syntheses. | - |
|  | 13d | Describe any methods used to synthesize results and provide a rationale for the choice(s). If meta-analysis was performed, describe the model(s), method(s) to identify the presence and extent of statistical heterogeneity, and software package(s) used. | - |
|  | 13e | Describe any methods used to explore possible causes of heterogeneity among study results (e.g. subgroup analysis, meta-regression). | - |
|  | 13f | Describe any sensitivity analyses conducted to assess robustness of the synthesized results. | - |
| Reporting bias assessment | 14 | Describe any methods used to assess risk of bias due to missing results in a synthesis (arising from reporting biases). | - |
| Certainty assessment | 15 | Describe any methods used to assess certainty (or confidence) in the body of evidence for an outcome. | - |
| **RESULTS** | | |  |
| Study selection | 16a | Describe the results of the search and selection process, from the number of records identified in the search to the number of studies included in the review, ideally using a flow diagram. | Page 5. Results. Results overview. From: “We retrieved 1507 publications in international (Medline/PubMed [n = 651]” to “Thus, we finally obtained a total of 58 publications”  Figure 1 (PRISMA flow diagram) |
|  | 16b | Cite studies that might appear to meet the inclusion criteria, but which were excluded, and explain why they were excluded. | - |
| Study characteristics | 17 | Cite each included study and present its characteristics. | Pages 5 and 6. Results. Results overview. From: “we finally obtained a total of 58 publications; of which, 38 (63.3%) were observational studies” to “Among the observational studies analyzed, 16 (43.2 %) had good compliance with STROBE guidelines”  Table 2 (Summary of the identified studies) |
| Risk of bias in studies | 18 | Present assessments of risk of bias for each included study. | Page 6. Results. Results overview. “We applied the STROBE criteria in 37 publications based on cohort observational designs and excluded a case-control study. Among the observational studies analyzed, 16 (43.2 %) had good compliance with STROBE guidelines”  Table 2 (Summary of the identified studies) column Compliance with the reporting guidelines |
| Results of individual studies | 19 | For all outcomes, present, for each study: (a) summary statistics for each group (where appropriate) and (b) an effect estimate and its precision (e.g. confidence/credible interval), ideally using structured tables or plots. | Table 2 (Summary of the identified studies) |
| Results of syntheses | 20a | For each synthesis, briefly summarise the characteristics and risk of bias among contributing studies. | - |
|  | 20b | Present results of all statistical syntheses conducted. If meta-analysis was done, present for each the summary estimate and its precision (e.g. confidence/credible interval) and measures of statistical heterogeneity. If comparing groups, describe the direction of the effect. | - |
|  | 20c | Present results of all investigations of possible causes of heterogeneity among study results. | - |
|  | 20d | Present results of all sensitivity analyses conducted to assess the robustness of the synthesized results. | - |
| Reporting biases | 21 | Present assessments of risk of bias due to missing results (arising from reporting biases) for each synthesis assessed. | - |
| Certainty of evidence | 22 | Present assessments of certainty (or confidence) in the body of evidence for each outcome assessed. | - |
| **DISCUSSION** | | |  |
| Discussion | 23a | Provide a general interpretation of the results in the context of other evidence. | Pages 9-14; discussion |
|  | 23b | Discuss any limitations of the evidence included in the review. | Page 12; discussion. Limitations and strengths. “…we acknowledge that we appraised a wide range of publications, including those with low reporting quality or a low level of evidence such as narrative reviews. We recognize that the latter might introduce the author’s bias, which might likewise affect the results and conclusions of our study” |
|  | 23c | Discuss any limitations of the review processes used. | Page 12; discussion. Limitations and strengths. “the inclusion criteria were limited to English and Spanish publications possibly resulting in the omission of critical publications in other languages. Finally, as we did not limit the study period, older studies included did not contemplate all the available therapeutic options” |
|  | 23d | Discuss implications of the results for practice, policy, and future research. | Page 13; discussion. Conclusions. “Our findings support ... The appraised evidence illustrates significant heterogeneity in the criteria used to define anti-TNF-α failure.”, “.most studies failed to consider patient-reported outcomes in assessing psoriasis treatment, which contrasts with recent recommendations on the inclusion of patient-reported HRQoL as a supporting criterion when considering clinical outcomes”, “our results also suggest that other factors should be considered, such as patients’ treatment preferences or adherence, comorbidities, or even hospital change that might influence anti-TNF-α adherence”, “our findings reflect a paradigm shift in the treatment sequence in the last ten years, incorporating new targets” |
| **OTHER INFORMATION** | | |  |
| Registration and protocol | 24a | Provide registration information for the review, including register name and registration number, or state that the review was not registered. | - |
|  | 24b | Indicate where the review protocol can be accessed, or state that a protocol was not prepared. | - |
|  | 24c | Describe and explain any amendments to information provided at registration or in the protocol. | - |
| Support | 25 | Describe sources of financial or non-financial support for the review, and the role of the funders or sponsors in the review. | Title page |
| Competing interests | 26 | Declare any competing interests of review authors. | Title page |
| Availability of data, code and other materials | 27 | Report which of the following are publicly available and where they can be found: template data collection forms; data extracted from included studies; data used for all analyses; analytic code; any other materials used in the review. | - |

Supplementary Table 3. List of search terms considered for the search

| **List of search terms considered for international databases (Pubmed/Medline and Cochrane)** | | |
| --- | --- | --- |
| **Disease** | **Anti-TNF-α** | **Therapeutic failure** |
| “Psoriasis”  “Psoriasis”[Mesh] | “Tumor necrosis factor inhibitors”  “TNF-inhibitors”  “TNF-α”  “TNF”  “anti-TNF”  “etanercept”  “adalimumab”  “infliximab”  “certolizumab” | “Failure”  “Switch”  “Switching”  “Transitioning”  “Second-line”  “lack of efficacy”  “loss of efficacy”  “lack of adherence”  “discontinuation”  “unmet need” |
| **List of search terms considered for national databases MEDES and IBECS** | | |
| **Disease** | **Anti-TNF-α** | **Therapeutic failure** |
| Psoriasis | “Inhibidor del factor de necrosis tumoral”  “inhibidor de TNF”  “TNF-α”  “TNF”  “anti-TNF”  “etanercept”  “adalimumab”  “infliximab”  “certolizumab” | “Fallo”  “Cambio”  “Transición”  “Segunda línea”  “Falta de eficacia”  “Pérdida de eficacia”  “Falta de adherencia”  “Discontinuación”  “Necesidad no cubierta” |
|  | | |

Supplementary Table 4. Search strategies

| **Search strategies used by database** | | | | |
| --- | --- | --- | --- | --- |
| **Database** | **Search Dates** | **Languages** | **Articles type** | **Search Strategy** |
| MEDLINE/Cochrane | No limitation | Full-text available in English/Spanish | Any | (“Psoriasis” OR “Psoriasis”[MeSH]) AND (“Tumor necrosis factor inhibitors” OR “TNF-inhibitors” OR “TNF-α” OR “TNF” OR “anti-TNF” OR “etanercept” OR “adalimumab” OR “infliximab” OR “certolizumab”) AND (“Failure” OR “Switch” OR “Switching” OR “Transitioning” OR “Second-line” OR “lack of efficacy” OR “loss of efficacy” OR “lack of adherence” OR “discontinuation” OR “unmet need”) |
| MEDES/IBECS | No limitation | Full-text available in English/Spanish | Any | Psoriasis AND Inhibidor del factor de necrosis tumoral  Psoriasis AND inhibidor de TNF  Psoriasis AND TNF-α  Psoriasis AND TNF  Psoriasis AND anti-TNF  Psoriasis AND etanercept  Psoriasis AND adalimumab  Psoriasis AND infliximab  Psoriasis AND certolizumab  Psoriasis AND Fallo  Psoriasis AND Cambio  Psoriasis AND Transición  Psoriasis AND Segunda línea  Psoriasis AND Falta de eficacia  Psoriasis AND Pérdida de eficacia  Psoriasis AND Falta de adherencia  Psoriasis AND Discontinuación  Psoriasis AND Necesidad no cubierta |

Supplementary Table 5. Gray literature sources

| **Source and search strategies used** | |
| --- | --- |
| **Source** | **Search strategy** |
| Google/Google Scholar | General search including English and Spanish terms such as psoriasis, anti-TNF-α, AND/OR “therapeutic failure.”  To identify publications focusing mainly on the criteria used to define anti-TNF-α and other issues related to the anti-TNF-α failure. |
| Websites of the main websites of national and international scientific societies (such as the AEDV, EADV, and the AAD. | Specific search to identify the most recent clinical practice guidelines and expert consensus. |
| Websites of the main websites for Health Technology Assessment (such as the NICE and the SEFH). | Specific search to identify relevant references (mainly evaluation and genesis reports for anti-TNF-α and anti-interleukin drugs, including biosimilars). |
| Abbreviations: AAD (American Academy of Dermatology); AEDV (Academia Española de Dermatología y Venereología); anti-TNF-α (tumor necrosis factor inhibitors); EADV (European Academy of Dermatology and Venereology); NICE (National Institute for Health and Care Excellence); SEFH (Sociedad Española de Farmacia Hospitalaria) | |

Supplementary Table 6. Description of the variables extracted in the included articles

| **Variables extracted in the included articles** | |
| --- | --- |
| Variables | Description |
| First author | First author’s name |
| Year of publication | Year of publication of the study |
| Period of the study | Time period in which the study was conducted |
| Type of publication | Design of the publication (observational retrospective, observational prospective, observational case stud, clinical trial, clinical practice guidelines, systematic revision, narrative revision) |
| Treatment arm characteristics | Type of anti-TNF-α treatment (etanercept, adalimumab, infliximab, and, certolizumab), number of patients in each arm, and percentage of men |
| Review outcome | Outcome extracted from the publication (criteria defining anti-TNF-α failure, reasons for treatment failure, therapeutic options used after anti-TNF-α failure, recommendations about therapeutic options used after anti-TNF-α failure, and main factors to consider when choosing a new therapy after anti-TNF-α failure) |
| Level of evidence | Level of evidence of publications according to the OCEBM scales[2] from 1a (systematic reviews of randomized clinical trials) to 5 (expert opinion without explicit critical appraisal). |
| Compliance with the reporting guidelines | For randomized clinical trials, number of items met/total items (%) out of the CONSORT statement for randomized clinical trials[3].  For observational cohort studies, number of items met/total items (%) out of the 22 essential points of the STROBE declaration for observational cohort studies[4]. |
| Abbreviations: anti-TNF-α (tumor necrosis factor inhibitors); CONSORT (Consolidated Standards of Reporting Trials); OCEBM (Oxford Centre for Evidence-Based Medicine); STROBE (Strengthening the Reporting of Observational studies in Epidemiology) | |

Supplementary Table 7. Summary of the studies included

| **Search strategies used by database** | | | | | |
| --- | --- | --- | --- | --- | --- |
| **Author, year of publication, and period of the study** | **Type of publication** | **Treatment arm characteristics** | **Review objective** | **Level of evidence** | **Compliance with the reporting guidelines** |
| **Arnold, 2016**  **(2003-2014)[5]** | Retrospective observational study | ADA: 137 patients; 60.6% men  ETA: 46 patients; 50.0% men  INF: 40 patients; 62.5% men | Reasons for anti-TNF-α treatment discontinuation | 4 | 19 (86.4%)  STROBE |
| **Amatore, 2019**  **(NA)[6]** | Clinical practice guidelines | NA | Recommendations on choice of treatment after anti-TNF-α failure | - | Does not apply as it has a different design from randomized clinical trials (CONSORT) or an observational cohort study (STROBE) |
| **Ayala, 2014**  **(NA)[7]** | Prospective observational study | INF: 38 patients; 73.7% men | Current criteria defining anti-TNF-α failure  Reasons for anti-TNF-α treatment discontinuation  Therapeutic options used after anti-TNF-α failure | 3 | 16 (72.7%)  STROBE |
| **Bagel, 2017**  **(2012-2015)[8]** | Prospective observational study | Unspecified anti-TNF-α: 64 patients; 62.5% men | Current criteria defining anti-TNF-α failure  Therapeutic options used after anti-TNF-α failure | 3 | 17 (77.3%)  STROBE |
| **Bardazzi, 2014**  **(2007-2012)[9]** | Retrospective observational study | ADA: 47 patients; 74.5% men  ETA: 45 patients; 55.6% men  INF: 33 patients; 54.5% men | Current criteria defining anti-TNF-α failure | 4 | 9 (40.9%)  STROBE |
| **Bayaraa, 2019**  **(2010-2017)[10]** | Retrospective observational study | INF: 48 patients; 77.1% men  ADA: 71 patients; 70.4% men | Reasons for anti-TNF-α treatment discontinuation  Therapeutic options used after anti-TNF-α failure | 4 | 12 (54.5%)  STROBE |
| **Bewley, 2011**  **(2008-2009)[11]** | Retrospective observational study | Unspecified anti-TNF-α:176 patients | Current criteria defining anti-TNF-α failure | 4 | 13 (59.1%)  STROBE |
| **Bhutani, 2011**  **(NA)[12]** | Retrospective observational study. Case study | ETA/ADA: 4 patients; 25.0% men | Therapeutic options used after anti-TNF-α failure | 4 | The evaluation of compliance with STROBE reporting guidelines does not apply as it has a different design from an observational cohort study |
| **Bissonnette, 2011**  **(NA-2008)[13]** | Clinical trial  Phase IV | ETA: 85 patients; 70.6% men | Current criteria defining anti-TNF-α failure  Therapeutic options used after anti-TNF-α failure | 2 | The evaluation of compliance with CONSORT reporting guidelines does not apply as it has a non-randomized design |
| **Bissonnette, 2015**  **(NA)[14]** | Prospective observational study | ADA: 67 patients; 73.1% men  INF: 14 patients; 78.6% men | Current criteria defining anti-TNF-α failure  Therapeutic options used after anti-TNF-α failure | 3 | 15 (68.2%)  STROBE |
| **Blauvelt, 2017**  **(2012-2013)[15]** | Randomized clinical trial  Phase III | ETA: 701 patients; 67.8% men | Current criteria defining anti-TNF-α failure  Therapeutic options used after anti-TNF-α failure | 2 | The evaluation of compliance with CONSORT reporting guidelines does not apply as it has a non-randomized design - |
| **Chiu, 2015**  **(2007-2013)[16]** | Retrospective observational study | ADA: 53 patients; 64.1% men | Current criteria defining anti-TNF-α failure | 3 | 15 (68.2%)  STROBE |
| **Clemmensen, 2010**  **(2009-NA)[17]** | Retrospective observational study | ADA/ETA: 108 patients; 53.7% men | Current criteria defining anti-TNF-α failure  Reasons for anti-TNF-α treatment discontinuation | 4 | 12 (54.5%)  STROBE |
| **Dávila, 2016**  **(2008-2013)[18]** | Prospective observational study | ADA: 598 patients; 62.5% men  ETA: 534 patients; 59.2% men  INF: 166 patients; 64.5% hombres | Reasons for anti-TNF-α treatment discontinuation | 3 | 20 (90.9%)  STROBE |
| **Di Lernia, 2013**  **(NA)[19]** | Retrospective observational study | Unspecified anti-TNF-α: 110 patients | Current criteria defining anti-TNF-α failure | 4 | 7 (31,8%)  STROBE |
| **Espósito, 2018**  **(2010-2014)[20]** | Retrospective observational study | Unspecified anti-TNF-α: 115 patients; 70.4% men | Therapeutic options used after anti-TNF-α failure | 4 | 14 (63.6%)  STROBE |
| **Espósito, 2013**  **(2007-2011)[21]** | Retrospective observational study | Unspecified anti-TNF-α: 650 patients; 65.5% men | Current criteria defining anti-TNF-α failure  Reasons for anti-TNF-α treatment discontinuation | 4 | 18 (81.8%)  STROBE |
| **Fonseca, 2014**  **(2005-2013)[22]** | Retrospective observational study | ADA: 35 patients; 77.1% men | Current criteria defining anti-TNF-α failure  Reasons for anti-TNF-α treatment discontinuation  Therapeutic options used after anti-TNF-α failure | 4 | 14 (63.6%)  STROBE |
| **Gerdes, 2018**  **(2013-2015)[23]** | Randomized clinical trial  Phase III | GP2015: 196 patients; 59.7% men  ETA: 301 patients; 63.8% men | Reasons for anti-TNF-α treatment discontinuation | 2 | The evaluation of compliance with CONSORT reporting guidelines does not apply as it has a non-randomized design - |
| **Gil-Sierra, 2020**  **(2010-2019)[24]** | Retrospective observational study | UST: 36 patients; 61.1% men | Therapeutic options used after anti-TNF-α failure | 4 | 11 (50,0%)  STROBE |
| **Gniadecki, 2011**  **(2007-2010)[25]** | Retrospective observational study | Unspecified anti-TNF-α: 747 patients; 65.5% men | Reasons for anti-TNF-α treatment discontinuation | 4 | 17 (77,3%)  STROBE |
| **Gottlieb, 2012**  **(2007-2009)[26]** | Prospective observational study | INF: 215 patients; 63.7% men | Current criteria defining anti-TNF-α failure  Therapeutic options used after anti-TNF-α failure | 3 | 18 (81.8%)  STROBE |
| **Griffiths, 2010**  **(2007-2009)[27]** | Clinical trial  Phase IV | ETA: 347 patients; 70.9% men | Current criteria defining anti-TNF-α failure  Therapeutic options used after anti-TNF-α failure | 3 | 22.5 (64,3%)  CONSORT |
| **Hercogova, 2019**  **(2016-2017)[28]** | Randomized clinical trial  Phase III | Biosimilar MSB11022: 203 patients; 67.0% men  ADA: 191 patients; 68.1% men | Current criteria defining anti-TNF-α failure | 2 | 27.5 (83.3%)  CONSORT |
| **Honda, 2017**  **(2010-2014)[29]** | Retrospective observational study | ADA: 91 patients; 71.4% men  INF: 59 patients; 78.0% men  UST: 125 patients; 72% men | Current criteria defining anti-TNF-α failure  Therapeutic options used after anti-TNF-α failure | 4 | 11 (50.0%)  STROBE |
| **Hu, 2017**  **(NA)[30]** | Narrative revision | NA | Recommendations about the election of treatment after anti-TNF-α failure | - | Does not apply as it has a different design -from randomized clinical trials (CONSORT) or an observational cohort study (STROBE) |
| **Iskandar, 2018**  **(2007-2016)[31]** | Prospective observational study | ETA: 104 patients; 47.1% men  ADA: 538 patients; 58.7% men | Therapeutic options used after anti-TNF-α failure | 3 | 21 (95.5%)  STROBE |
| **Lebwohl, 2018**  **(2015-2016)[32]** | Randomized clinical trial Phase III | ETA: 170 patients; 74.7% men  CZP 200mg: 165 patients; 68.5% men  CZP 400mg: 167 patients; 64.1% men | Current criteria defining anti-TNF-α failure  Therapeutic options used after anti-TNF-α failure | 2 | 28.5 (83.8%)  CONSORT |
| **Lunder, 2019**  **(2005-2018)[33]** | Retrospective observational study | ADA: 750 patients; 62.8% men  ETA: 66 patients; 57.6% men  INF: 77 patients; 57.1% men | Reasons for anti-TNF-α treatment discontinuation | 4 | 18 (81.8%)  STROBE |
| **Marty-simmons, 2009**  **(NA)[34]** | Clinical trial  Phase IV | ADA: 5 patients; 60% men | Current criteria defining anti-TNF-α failure  Therapeutic options used after anti-TNF-α failure | 3 | The evaluation of compliance with CONSORT reporting guidelines does not apply as it has a non-randomized design - |
| **Menter, 2008**  **(NA)[35]** | Randomized clinical trial  Phase III | ADA: 814 patients; 67.1% men | Current criteria defining anti-TNF-α failure | 2 | 24.5 (81.7%)  CONSORT |
| **Menter, 2016**  **(2007-2013)[36]** | Prospective observational study | INF: 63 patients; 61.9% men  ADA: 402 patients; 57.2% men  ETA: 289 patients; 55.0% men | Reasons for anti-TNF-α treatment discontinuation | 3 | 19 (86.4%)  STROBE |
| **Ortonne, 2011**  **(NA)[37]** | Randomized clinical trial Phase III | ADA: 730 patients; 68.6% men | Reasons for anti-TNF-α treatment discontinuation  Therapeutic options used after anti-TNF-α failure | 3 | The evaluation of compliance with CONSORT reporting guidelines does not apply as it has a non-randomized design - |
| **Piaserico, 2014**  **(2005-2009)[38]** | Retrospective observational study | Unspecified anti-TNF-α: 105 patients; 64.8% men | Current criteria defining anti-TNF-α failure  Reasons for anti-TNF-α treatment discontinuation  Therapeutic options used after anti-TNF-α failure | 4 | 15 (68.2%)  STROBE |
| **Pink, 2009**  **(2008)[39]** | Retrospective observational study | Unspecified anti-TNF-α: 97 patients | Current criteria defining Anti-TNF-α failure | 4 | 10 (45.5%)  STROBE |
| **Pitarch, 2008**  **(NA)[40]** | Prospective observational study | Unspecified anti-TNF-α: 8 patients; 87.5% men | Current criteria defining Anti-TNF-α failure  Therapeutic options used after anti-TNF-α failure | 3 | 8 (36.4%)  STROBE |
| **Puig, 2019**  **(2012-2014)[41]** | Prospective observational study | Unspecified anti-TNF-α: 371 patients; 65.0% men | Reasons for anti-TNF-α treatment discontinuation | 3 | 17 (77.3%)  STROBE |
| **Puig, 2013**  **(NA)[42]** | Clinical practice guidelines | NA | Current criteria defining Anti-TNF-α failure  Recommendations about the election of treatment after anti-TNF-α failure | - | Does not apply as it has a different design -from randomized clinical trials (CONSORT) or an observational cohort study (STROBE) |
| **Puig, 2008**  **(2004-2007)[43]** | Retrospective observational study | INF: 43 patients; 65.1% men | Current criteria defining Anti-TNF-α failure | 4 | 11 (50.0%)  STROBE |
| **Reich, 2005 (EXPRESS)**  **(NA)[44]** | Randomized clinical trial  Phase III | INF: 301 patients; 68.8% men | Current criteria defining Anti-TNF-α failure | 2 | 21 (65.6%)  CONSORT |
| **Reich, 2019 (IMMVent)**  **(2016-2017)[45]** | Randomized clinical trial  Phase III | ADA: 304 patients; 70.0% men | Current criteria defining Anti-TNF-α failure  Therapeutic options used after anti-TNF-α failure | 2 | 25.5 (79.7%)  CONSORT |
| **Reich, 2019 (RESURFACE)**  **(2012-2015)**  **(2013-2015)[46]** | Randomized Clinical trial  Phase III | ETA: 313 patients; 70.9% men | Current criteria defining Anti-TNF-α failure  Therapeutic options used after anti-TNF-α failure | 2 | 32 (97,0%)  CONSORT |
| **Reich, 2017 (VOYAGEII)**  **(2014-2016)[47]** | Randomized clinical trial  Phase III | ADA: 248 patients; 68.5% men | Current criteria defining anti-TNF-α failure  Therapeutic options used after anti-TNF-α failure | 2 | 21.5 (67.2%)  CONSORT |
| **Roche, 2018**  **(2005-2016)[48]** | Retrospective observational study | Unspecified anti-TNF-α: 269 patients; 66.5% men | Reasons for anti-TNF-α treatment discontinuation | 4 | 11 (50.0%)  STROBE |
| **Ruiz Genao, 2020**  **(2008-2010)**  **(2011-2014)**  **(2015-2018)[49]** | Prospective observational study | Unspecified anti-TNF-α: 581 patients (2008-2010); 61.8% men  Unspecified anti-TNF-α: 190 patients (2011-2014); 61.1% men  Unspecified anti-TNF-α (2015-2018): 304 patients; 53.9% men | Reasons for anti-TNF-α treatment discontinuation | 3 | 17 (77.3%)  STROBE |
| **Smith, 2017**  **(2010-2015)[50]** | Retrospective observational study | Unspecified anti-TNF-α: 116 patients; 43.1% men | Reasons for anti-TNF-α treatment discontinuation  Therapeutic options used after anti-TNF-α failure | 4 | 13 (59.1%)  STROBE |
| **Smith, 2017[51]** | Clinical practice guidelines | NA | Recommendations about the election of treatment after anti-TNF-α failure  Factors to consider when choosing a new therapy after anti-TNF-α failure | - | Does not apply as it has a different design -from randomized clinical trials (CONSORT) or an observational cohort study (STROBE) |
| **Spertino, 2013**  **(2004-2012)[52]** | Retrospective observational study | INF: 56 patients; 62.5% men | Current criteria defining Anti-TNF-α failure  Therapeutic options used after anti-TNF-α failure | 4 | 17 (77.3%)  STROBE |
| **Strober, 2011**  **(2008-2009)[53]** | Randomized clinical trial Phase III | ETA: 152 patients; 59.9% men | Current criteria defining Anti-TNF-α failure  Therapeutic options used after anti-TNF-α failure | 2 | The evaluation of compliance with CONSORT reporting guidelines does not apply as it has a non-randomized design |
| **Takahashi, 2015**  **(2011-2013)[54]** | Prospective observational study | ADA: 18 patients; 90% men | Current criteria defining Anti-TNF-α failure | 3 | 10 (45.5%)  STROBE |
| **Talamonti, 2018**  **(2010-2015)[55]** | Retrospective observational study | ADA: 262 patients; 63.3% men | Current criteria defining Anti-TNF-α failure  Therapeutic options used after anti-TNF-α failure | 4 | 16 (72.7%)  STROBE |
| **Umezawa, 2013**  **(NA)[56]** | Prospective observational study | INF: 38 patients; 78.9% men  ADA: 59 patients; 67.8% men | Current criteria defining Anti-TNF-α failure | 3 | 10 (45.5%)  STROBE |
| **van der Reeck, 2014**  **(2007-2012)[57]** | Prospective observational study | ADA: 116 patients; 55.2% men | Reasons for anti-TNF-α treatment discontinuation | 3 | 18 (81.8%)  STROBE |
| **Van Lümig, 2010**  **(NA)[58]** | Prospective observational study | ADA: 30 patients; 63.3% men | Current criteria defining Anti-TNF-α failure  Therapeutic options used after anti-TNF-α failure | 3 | 14 (63.6%)  STROBE |
| **Vender, 2011[59]** | Clinical trial  Phase IV | ETA: 10 patients | Current criteria defining Anti-TNF-α failure  Therapeutic options used after anti-TNF-α failure | 3 | The evaluation of compliance with CONSORT reporting guidelines does not apply as it has a non-randomized design |
| **Viguier, 2013**  **(NA)[60]** | Retrospective observational study  Survey | Unspecified anti-TNF-α: 22 patients; 45.0% men | Current criteria defining anti-TNF-α failure | 3 | 9 (40.9%)  STROBE |
| **Warren, 2019**  **(2013-2016)[61]** | Clinical trial  Phase IV | Unspecified anti-TNF-α: 235 patients; 56.2% men | Current criteria defining anti-TNF-α failure  Therapeutic options used after anti-TNF-α failure | 3 | 25 (78.1%)  CONSORT |
| **Yeung, 2013**  **(2010-2011)[62]** | Prospective observational study  Survey | Unspecified anti-TNF-α: 1095 patients; 48.6% men | Reasons for anti-TNF-α treatment discontinuation | 3 | 19 (86.4%)  STROBE |

Abbreviations: ADA (adalimumab); anti-TNF-α (tumor necrosis factor inhibitors); CONSORT (Consolidated Standards of Reporting Trials); CZP (certolizumab pegol); ETA (etanercept); INF (infliximab); NA (non-available); STROBE (Strengthening the Reporting of Observational studies in Epidemiology); UST (ustekinumab); CONSORT report criteria were assessed in randomized clinical trials whereas STROBE criteria in publications with an observational cohort design

Supplementary Table 8. Criteria reported by each studio for primary and secondary fail

| **Search strategies used by database** | |  | |  |
| --- | --- | --- | --- | --- |
| **Author, year of publication, and period of the study** | **Primary failure criteria** | | **Secondary failure criteria** | |
| **Ayala, 2014**  **(NA)[7]** | PASI75 | |  | |
| **Bagel, 2017**  **(2012-2015)[8]** | - | | PASI90/PGA 0-1 | |
| **Bardazzi, 2014**  **(2007-2012)[9]** | PASI50 | |  | |
| **Bewley, 2011**  **(2008-2009)[11]** | PASI75 and HRQoL | |  | |
| **Bissonnette, 2011**  **(NA-2008)[13]** | PASI90/PGA 0-1 | | PASI90/PGA 0-1 | |
| **Bissonnette, 2015**  **(NA)[14]** | PASI90/PGA 0-1 | | PASI90/PGA 0-1 | |
| **Blauvelt, 2017**  **(2012-2013)[15]** | PASI90/PGA 0-1 | |  | |
| **Chiu, 2015**  **(2007-2013)[16]** | PASI75 | |  | |
| **Clemmensen, 2010**  **(2009-NA)[17]** | PASI50 and HRQoL | |  | |
| **Di Lernia, 2013**  **(NA)[19]** | PASI50 | | PASI50 | |
| **Espósito, 2013**  **(2007-2011)[21]** | PASI50 | | PASI50 | |
| **Fonseca, 2014**  **(2005-2013)[22]** | PASI50 | | PASI50 | |
| **Griffiths, 2010**  **(2007-2009)[27]** | Non-objective | |  | |
| **Gottlieb, 2012**  **(2007-2009)[26]** | Non-objective | |  | |
| **Hercogova, 2019**  **(2016-2017)[28]** | PASI50 | |  | |
| **Honda, 2017**  **(2010-2014)[29]** | PASI50 | | PASI50 | |
| **Lebwohl, 2018**  **(2015-2016)[32]** | PASI50 | |  | |
| **Marty-simmons, 2009**  **(NA)[34]** | PASI50 | |  | |
| **Menter, 2008**  **(NA)[35]** | - | | PASI50 | |
| **Piaserico, 2014**  **(2005-2009)[38]** | PASI75 | | PASI75 | |
| **Pink, 2009**  **(2008)[39]** | PASI50 | |  | |
| **Pitarch, 2008**  **(NA)[40]** | PASI75 | |  | |
| **Puig, 2013**  **(NA)[42]** | PASI50 | | PASI50 | |
| **Puig, 2008**  **(2004-2007)[43]** | PASI50 | | PASI50 | |
| **Reich, 2005 (EXPRESS)**  **(NA)[44]** | PASI50 | |  | |
| **Reich, 2019 (IMMVent)**  **(2016-2017)[45]** | PASI50 | |  | |
| **Reich, 2019 (RESURFACE)**  **(2012-2015)**  **(2013-2015)[46]** | PASI50 | |  | |
| **Reich, 2017 (VOYAGEII)**  **(2014-2016)[47]** | PASI90/PGA 0-1 | |  | |
| **Spertino, 2013**  **(2004-2012)[52]** | PASI50 | | PASI50 | |
| **Strober, 2011**  **(2008-2009)[53]** | PASI90/PGA 0-1 | | Non-specific | |
| **Takahashi, 2015**  **(2011-2013)[54]** | PASI50 | |  | |
| **Talamonti, 2018**  **(2010-2015)[55]** | PASI50 | | PASI50 | |
| **Umezawa, 2013**  **(NA)[56]** | PASI50 | | Non-specific | |
| **Van Lümig, 2010**  **(NA)[58]** | PASI50 | | PASI50 | |
| **Vender, 2011[59]** | PASI90/PGA 0-1 | |  | |
| **Viguier, 2013**  **(NA)[60]** | PASI50 | | PASI50 | |
| **Warren, 2019**  **(2013-2016)[61]** | PASI75 and HRQoL | | PASI75 | |

Abbreviations: PASI (Psoriasis Area and Severity Index); HRQOL (Health-related quality of life); NA (non-available)

Supplementary Figure 1. Treatment after anti-TNF-α failure reported over time


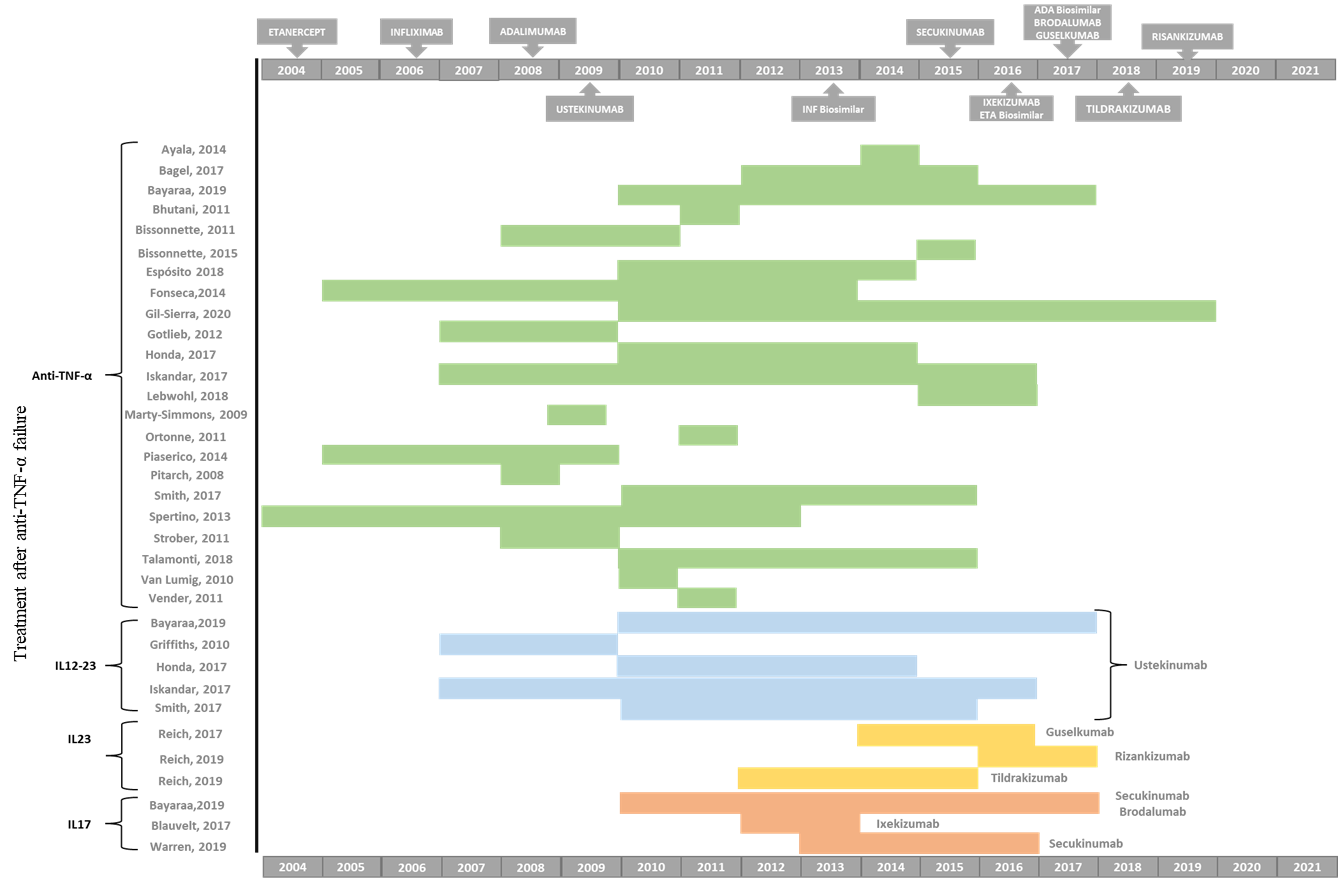


The ordinate axis shows articles grouped by the treatment chosen after anti-TNF-α failure reported, whereas the abscissa axis shows the period in which they were conducted. Band colors represent the treatment chosen after anti-TNF-α failure in the studies: green = another anti-TNF-α; blue = IL12-23; yellow= IL23; orange = IL17. Abbreviations: IL (interleukin); anti-TNF-α (tumor necrosis factor inhibitors); we did not include the IL-17 inhibitor, bimekizumab, in the search since it had not yet been approved at the time of the study.

**References**
